# Role of* Burkholderia pseudomallei* Sigma N2 in Amino Acids Utilization and in Regulation of Catalase E Expression at the Transcriptional Level

**DOI:** 10.1155/2015/623967

**Published:** 2015-12-22

**Authors:** Duong Thi Hong Diep, Nguyen Thi Thanh Phuong, Mya Myintzu Hlaing, Potjanee Srimanote, Sumalee Tungpradabkul

**Affiliations:** ^1^Department of Biochemistry, Faculty of Science, Mahidol University, Bangkok 10400, Thailand; ^2^Department of Clinical Microbiology, Faculty of Medical Technology, Mahidol University, Salaya 73170, Thailand; ^3^Graduate Programme in Biomedical Sciences, Faculty of Allied Health Science, Thammasat University, Rungsit 12121, Thailand

## Abstract

*Burkholderia pseudomallei* is the causative agent of melioidosis. The complete genome sequences of this pathogen have been revealed, which explain some pathogenic mechanisms. In various hostile conditions, for example, during nitrogen and amino acid starvation, bacteria can utilize alternative sigma factors such as RpoS and RpoN to modulate genes expression for their adaptation and survival. In this study, we demonstrate that mutagenesis of* rpoN2*, which lies on chromosome 2 of* B. pseudomallei* and encodes a homologue of the sigma factor RpoN, did not alter nitrogen and amino acid utilization of the bacterium. However, introduction of* B. pseudomallei rpoN2* into* E. coli* strain deficient for* rpoN* restored the ability to utilize amino acids. Moreover, comparative partial proteomic analysis of the* B. pseudomallei* wild type and its* rpoN2* isogenic mutant was performed to elucidate its amino acids utilization property which was comparable to its function found in the complementation assay. By contrast, the* rpoN2* mutant exhibited decreased* katE* expression at the transcriptional and translational levels. Our finding indicates that* B. pseudomallei* RpoN2 is involved in a specific function in the regulation of catalase E expression.

## 1. Introduction

Melioidosis is an endemic disease in Southeast Asia and northern Australia. The causative agent of melioidosis in humans is* Burkholderia pseudomallei* which is a facultative intracellular pathogen. This organism is a polar flagellated Gram negative bacterium that can infect both humans and animals [1–3]. The mechanism by which* B. pseudomallei* causes melioidosis and its virulence is partly understood. The major regulatory control mechanisms for the expression of genes including virulent genes are the sigma factors. There are two major families of sigma factors which are sigma 70 (RpoD) and sigma 54 (RpoN) [4]. Sigma 54 is required only for a specific metabolic pathway such as nitrogen utilization and amino acids synthesis [5]. Moreover, sigma 54 regulates the transcription of virulence associated genes including pili, flagella, and alginate biosynthesis operons in* Pseudomonas aeruginosa* and* Vibrio* species [6–8].

The analysis of* B. pseudomallei* genome has been identified in two copies of* rpoN* genes in two genomic locations,* rpoN1* on chromosome 1 and* rpoN2* on chromosome 2. To date, nothing is known at the molecular level regarding the function of sigma 54 (RpoN) in* B. pseudomallei*. Therefore, it was of interest to determine whether* B. pseudomallei* RpoN1 or RpoN2 is involved in nitrogen and amino acids utilization. To investigate this, the defined* B. pseudomallei* strain 844* rpoN1* and* rpoN2* knockout mutants were constructed. However, only* rpoN2* knockout mutant was successfully constructed. The role of RpoN2 in nitrogen and amino acids utilization was examined and compared to that of* E. coli* lacking RpoN by complementation assay in* rpoN* mutant derivative* E. coli* JKD 814. A comparative proteomic analysis of the* B. pseudomallei* wild type and its* rpoN2* isogenic mutant was performed to elucidate its amino acids utilization property. Moreover, we have identified RpoN2 specific function and found that it is involved in regulation of catalase E expression both at the transcriptional and at the translational levels.

## 2. Materials and Methods

### 2.1. Bacterial Strain and Growth Conditions

The bacterial strains used are listed in Table 1.* B. pseudomallei* is routinely maintained in Luria-Bertani (LB) medium.* Pseudomonas* agar base supplemented with SR103E (Cetrimide, Fucidin, and Cephaloridine) from Oxoid was used after conjugation as selective medium to inhibit growth of* E. coli*. Ashdown agar plate was also used for* B. pseudomallei* specific selective medium. All cultures were grown at 37°C in an aerobic condition with 250 rpm shaking. Tetracycline (60 *μ*g/mL) and chloramphenicol (40 *μ*g/mL) were added to media when required.

### 2.2. Construction of* B. pseudomallei rpoN2* Mutant and Its Complemented Strain

*rpoN2* knockout mutant (Table 1) was created to be pKRpoN2 according to a previously described procedure [9]. pKRpoN2 was constructed by transferring the 363 bp partial digested* Pst*I fragment from genomic DNA of* B. pseudomallei* into the mobilizable suicide vector pKNOCK-Tc [10]. The constructed* B. pseudomallei rpoN2* mutant was analyzed by Southern blot analysis and PCR as described elsewhere [11]. To confirm that all changes in phenotypes were caused by the disruption of* rpoN2* and were not due to polar effects on downstream genes, a plasmid (pBSDRpoN2) containing the complete full-length* rpoN2* coding sequence under control of the* lac*Z and* cat* promoters was constructed and transferred into the* B. pseudomallei* mutant strains for complementation analysis. Likewise, pTOPO::*rpoN2*_Bp_ and pTOPO::*rpoN2*_Bp799_ were constructed and used to complement into JKD 814* E. coli rpoN* negative mutant. All plasmids constructed are listed in Table 1.

### 2.3. Nitrogen and Amino Acids Utilization Tests

Nitrogen and amino acids utilization tests were performed in MM9 salts minimal agar containing either 20 mM ammonium chloride (NH_4_Cl) or other alternative nitrogen sources such as arginine, glutamine, glycine, histidine, lysine, methionine, phenylalanine, tryptophan, and valine at 5 mM concentration. 10 *μ*L of overnight growth of the desired bacterial strains (*A*_600_ = 1) was inoculated onto the above agar medium and incubated at 37°C. Growths of the bacterial colony were observed daily for five consecutive days.

Statistical measurements of all assays were carried out in three separate times. The results were expressed as the mean ± standard deviation of days of growth. The significance of differences in nitrogen and amino acids utilization of bacterial strains was analyzed by Student's paired* t*-test (2-tailed) using SPSS statistical software program.

### 2.4. Protein Extraction and Two-Dimensional Gel Electrophoresis (2DE)

Bacterial cultures were grown until early stationary phase. Proteins were extracted using 500 *μ*L lysis buffer (8 M urea, 4% w/v CHAPS, 2 mM TBP, 1% v/v IPG buffer, pH 4–7) (Amersham Biosciences, Uppsala, Sweden) and 1% v/v protease inhibitor cocktail set II (Calbiochem, La Jolla, CA). The supernatant after cells lysis was transferred into clean microcentrifuge tubes and stored at −80°C until use. Protein concentrations were determined using* RC DC* protein assay kit (Bio-Rad, Hercules, CA) as previously described [12]. For 2DE, the first-dimensional isoelectric focusing (IEF) was carried out using 500 *μ*g protein samples with the rehydration buffer (8 M urea, 2% w/v CHAPS, 20 mM DTT, and 1% v/v IPG buffer, pH 4–7) adjusted to 350 *μ*L total volume. Precast 18-cm Immobiline DryStrip with a linear pH 4–7 was used with the IPGphor II system (Amersham Biosciences) to perform IEF. The strips were rehydrated with the protein samples for 12 h at 20°C following three voltage steps as previously described [12]. All profiles were controlled at the current 50 *μ*A/strip. The IPG strips were then equilibrated and transferred to the second-dimensional SDS-PAGE using 12.5% polyacrylamide gel. SDS-PAGE was performed at 4°C with the constant electrical current at 10 mA/gel. Protein spots were visualized by Coomassie Brilliant Blue G-250 (CBBG-250) staining and gels were scanned with an ImageMaster Scanner (Amersham Biosciences). Image analysis was performed using PDQuest software version 7.1 (Bio-Rad). Images from three independent cultures were compared. A master gel used for spot matching process was created from a wild type 2D gel. The master gel was then used for matching of the corresponding protein spots between 2D gels. The relative intensity of each protein spot was determined by normalizing to the total intensity of the gel. Protein expression with intensity representing at least 3-fold difference with *P* < 0.05 was considered in this analysis. The biosynthesis pathways such as cysteine synthesis, histidine synthesis, purine metabolism, and pentose phosphate pathway were performed by using KEGG database [13].

### 2.5. Protein Extraction and Activity Staining for Catalase

Bacterial cultures were grown in various growth phase (12, 24, 48, and 72 hours) conditions. The bacterial pellets were lysed on ice by sonication in one-tenth of the original culture volume of phosphate buffer (5 mM potassium phosphate, pH 7.0, 5 mM EDTA, 10% glycerol, and 25 mM phenylmethylsulfonyl fluoride). Proteins were extracted as previously described [14]. The total protein concentration in each sample was determined with Bradford Reagent (Sigma Chemical, St. Louis, MO).

Catalase activities present in crude extracts of* B. pseudomallei* cells were determined by loading 20 *μ*g of protein in 12% nondenaturing polyacrylamide gel. After polyacrylamide gel electrophoresis, the gels were washed three times with PBS (20 min each) to remove surface attached buffer ions as previously described [14]. Immediately, the gels were incubated with a solution of 2% (w/v) ferric chloride-potassium ferricyanide, until the gel was stained green.

### 2.6. RNA Isolation, cDNA Synthesis, and Relative Gene Expression Analysis

RNA extraction procedures were performed using TRIzol reagent (Invitrogen, USA). 1 mL of various growth phase cultures (24, 48, and 72 hours) was harvested and collected by centrifugation. RNA extraction was carried out following the manufacturer's instructions. Each RNA sample was treated with RQ1 RNase-free DNase (Promega, USA) and tested for DNA contamination as previously described [11]. Measurement of RNA concentration was performed using Nanodrop 2000 (Thermo Fisher Scientific, USA). cDNA synthesis was performed using Superscript III Reverse Transcriptase First Strand cDNA synthesis kit (Invitrogen, USA) following the manufacturer's instructions.

Relative quantification real-time PCR for the* katE* gene was performed using Rotor-Gene 3000 (Corbett Research, Australia). Primers (*katE*-F primer 5′-TCT ACA CCG ACG AGG GCA AC-3′ and* katE-*R primer 5′-TTC CTC CGG AAT CAG CTT GG-3′) were designed using Primer3 software [19], and the reactions were quantified using SYBR GreenER qPCR SuperMix Universal (Invitrogen, USA). All reactions were programmed with dissociation curve analysis to prevent nonspecific and primer-dimer formation as previously described [20]. Gene encoding 23s rRNA was used as a reference control for normalization. The results were analyzed using the comparative Ct method or ΔΔCt method (Applied Biosystems).

Statistical analysis of this study was performed from at least 3 independent experiments, each carried out in duplicate or triplicate. Values were presented as means ± standard error. Statistical significance of differences between the two means was calculated using SigmaStat 3.5 software and evaluated by Student's *t*-test and *P* value <0.01 was considered significant.

## 3. Results

### 3.1. Nitrogen and Amino Acids Utilization Tests in* Escherichia coli*

In order to determine a constructed* rpoN2* mutant whether RpoN2 is essential for the* B. pseudomallei* growth in the absence of amino acid supplementation, we compared the growth of the wild type* rpoN2* isogenic mutant and the* rpoN2* mutant carrying* rpoN2-*complementing plasmid. No differences in growth were observed among the strains (data not shown). These results indicate that* rpoN2* may be either not necessary or lacking the functions for amino acid utilization phenotypes.

To determine whether the* rpoN2* is not necessary or lacks the functions in amino acid utilization in* B. pseudomallei*, we performed the complementation assay by transforming full-length* B. pseudomallei rpoN2* (TOPO::*rpoN2*_Bp_ plasmid DNA) and 799-fragment* rpoN2* (TOPO::*rpoN2*_Bp799_ plasmid DNA) into* E. coli* JKD 814 which lacks of* rpoN* and then compared with* E. coli* JKD 814 complemented with TOPO::*rpoN*_Ec_ plasmid DNA using as a positive control. 10 *μ*L (*A*_600_ = 1) of bacterial culture was inoculated on M9 minimal agar containing each amino acid and incubated at 37°C for 5 days.* E. coli rpoN* mutant JKD 814 and* E. coli* wild type JM 109 were negative and positive controls, respectively. The experiments were carried out in three biological replicates. The differences in utilization of each amino acid for each construct were compared to that of JKD 814 and analyzed using Student's paired *t*-test.* B. pseudomallei* wild type strain 844 showed similar colony growth rate to* E. coli* wild type in the utilization of histidine as sole nitrogen source. Like* E. coli* wild type,* B. pseudomallei* also grew on M9 minimal agar supplemented with NH_4_Cl, lysine, and tryptophan but the growths were approximately one-day delay (Table 2). Although the growth of* B. pseudomallei* could be investigated on arginine and valine sole nitrogen source, the colonies were not observed until after day four of incubation. On the other hand,* B. pseudomallei* wild type utilized glutamine and phenylalanine faster than* E. coli* wild type. However, unlike* E. coli* wild type,* B. pseudomallei* could not utilize glycine as a growth substrate.

The* rpoN* mutant* E. coli* JKD 814 and JKD 814 harboring either pCR 2.1-TOPO vector alone or plasmid containing only partial ORF of* rpoN2* (TOPO::*rpoN2*_Bp799_ [799 bp]) exhibited no growth on minimal media supplemented with all nitrogen sources tested in this study after 5 days of incubation (Table 2).

Although the delay in growth was demonstrated, when TOPO::*rpoN*_Ec_ recombinant plasmid which contained entire ORF of* E. coli rpoN* was complemented into JKD 814* E. coli rpoN* mutant, the growth phenotypes were able to restore all tested amino acids except methionine and valine (Table 2). In contrast, the mutant JKD 814 complemented TOPO::*rpoN*_Ec_ strain exhibited faster growing on the simple nitrogenous compound NH_4_Cl compared to the* E. coli* wild type. Similar growth patterns were also observed in the mutant JKD 814 complemented with TOPO::*rpoN2*_Bp_ with the exception of methionine. Moreover, in minimal media supplemented with sole lysine, the mutant JKD 814 complemented with TOPO::*rpoN2*_Bp_ appeared to grow slower than the mutant complemented with TOPO::*rpoN*_Ec_ and the* E. coli* wild type.

Compared to* B. pseudomallei* wild type strain 844,* rpoN* mutant JKD 814 complemented with TOPO::*rpoN2*_Bp_ grew slightly faster on M9 minimal media supplemented with NH_4_Cl, arginine, and tryptophan while it grew slower in glutamine, histidine, and phenylalanine. No difference in growth rate was inspected when lysine was used as a sole nitrogen source. Moreover, the valine utilization could not be restored in the* rpoN2*_Bp_ complemented strain. Surprisingly, the* rpoN* mutant JKD 814 complemented with TOPO::*rpoN2*_Bp_ was able to grow on media supplemented with glycine and methionine which were the characteristic of* E. coli* wild type and not of* B. pseudomallei* wild type strain 844 phenotype (Table 2).

The overall results suggested that any difference in nitrogen utilization observed in this study was mediated by RpoN from either* E. coli* or* B. pseudomallei*. Therefore, we have demonstrated that* B. pseudomallei rpoN2* has full functions in regulation of nitrogen and amino acids utilization.

### 3.2. Partial Proteomic Analysis of* B. pseudomallei rpoN2* Mutant Compared with Its Wild Type

**Figure 1:**
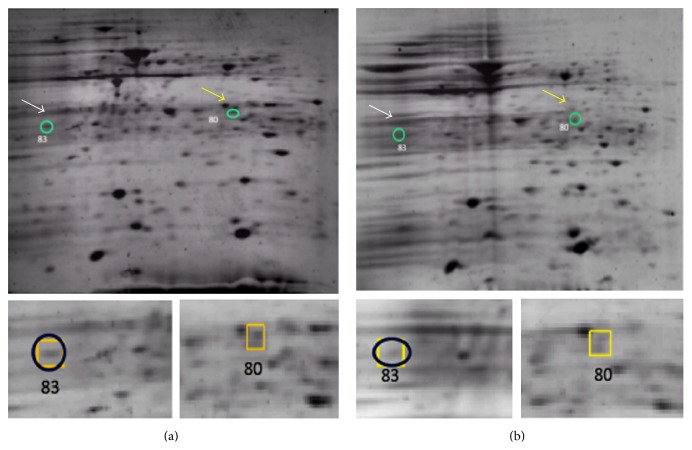
2D-gel electrophoresis of* B. pseudomallei* wild type (a) and* rpoN2* mutant (b) obtained from stationary phase of growth. PDQuest program was used for analysis of the group's sample of wild type and mutant compared with reference map of* B. pseudomallei*. The circles indicate spot numbers 80 (CysM) and 83 (HisA) that are downregulated in* rpoN2* mutant strain.

**Figure 2:**
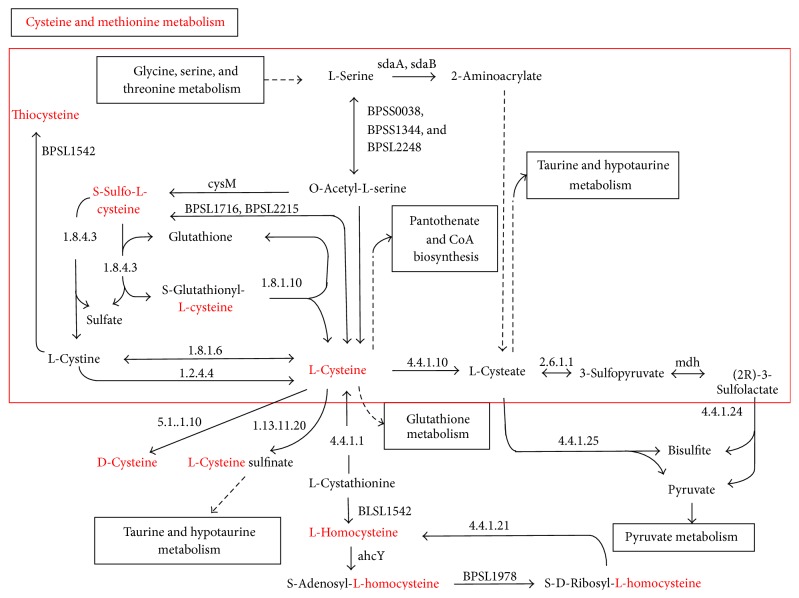
Cysteine and methionine metabolisms. The square is a pathway synthesis from glycine, serine, and threonine metabolisms to L-cysteine. http://www.genome.jp/kegg.

**Figure 3:**
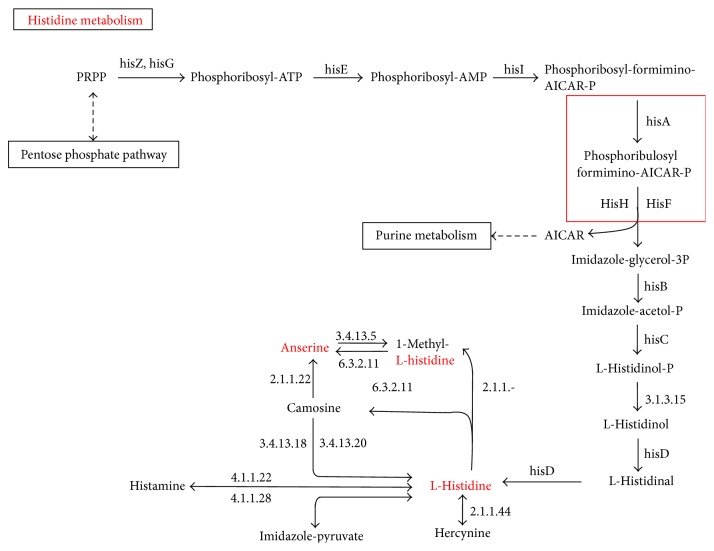
Histidine metabolism. The square is the phosphoribosyl formimino-5-aminoimidazole carboxamide ribotide isomerase (HisA). http://www.genome.jp/kegg.

A comparison of proteomic expressions in wild type and* rpoN2* mutant strains of* B. pseudomallei* was set as the cutoff to be a threefold difference between the wild type and* rpoN2* mutant. The results for proteins located in pH range 4.5 to 7 and molecular weight 20–75 kDa are shown in Figure 1. The identity of proteins was determined using the reference map from the wild type analysis [18]. A total of 21 spots were identified. The upregulated proteins in the* rpoN2* mutant strain included 14 proteins and the downregulated proteins in* rpoN2* mutant included 7 proteins as indicated in Figures 1(a) and 1(b). The numbers of each spot are referred to the same numbers as mentioned in the* B. pseudomallei* wild-type proteome reference map [18] which was used to study the RpoS regulon of* B. pseudomallei* [21]. The 7 proteins are downregulated in* rpoN2* mutant including proteins involved in energy metabolism, lipid metabolism, transcription, and several hypothetical proteins of unknown function. In addition, 14 proteins appeared to be upregulated in* rpoN2* mutant, the majority of which are involved in carbohydrate metabolism, posttranslation modification, cell envelope biogenesis, and outer membrane formation as summarized in Table 3. In order to identify the enzymes responsible for nitrogen utilization and amino acid synthesis and uptake, we found phosphoribosyl formimino-5-aminoimidazole carboxamide ribotide isomerase (HisA) (spot number 83) and cysteine synthase (CysM) (spot number 80) that are involved in histidine and cysteine synthesis, respectively (Figures 1(a) and 1(b)). However, both histidine and cysteine biosynthetic pathway can produce these amino acids from several starting points using both* de novo* synthesis and the intermediates in other biosynthetic pathways (Figures 2 and 3) [13]. Therefore,* B. pseudomallei* HisA and CysM are not essential for production of histidine and cysteine. Our partial proteomic analysis supported the function of RpoN2 that is not essential for nitrogen and amino acids utilization.

### 3.3. Positive Regulation of RpoN2 on* katE *in* B. pseudomallei*

**Figure 4:**
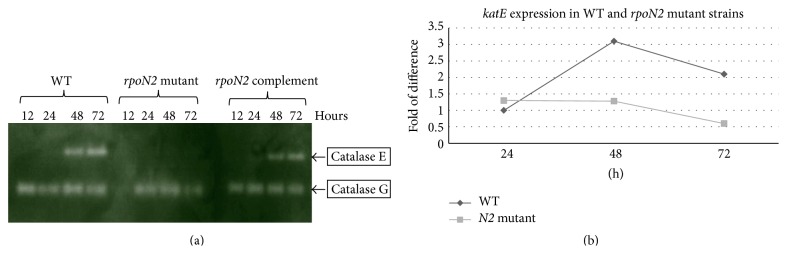
(a) Zymography of catalase activities during various stages.* B. pseudomallei* PP844 wild type (WT), the* rpoN2* isogenic mutant (*rpoN2* mutant), and the* rpoN2* mutant carrying the* rpoN2-*complementing plasmid (*rpoN2* complement) were grown aerobically in LB medium for 12, 24, 48, and 72 hours. The extracted cells (15 *μ*g of protein) were prepared for electrophoresis in 10% nondenaturing polyacrylamide gel and stained for catalase activity in a solution of 2% (w/v) ferric chloride-potassium ferric cyanide. (b) Relative quantification real-time RT-PCR (qRT-PCR) for* B. pseudomallei katE* expression was compared between the* rpoN2* isogenic mutant (*N2* mutant) and the wild type PP844 (WT) at various stages (24, 48, and 72 hours) of growth. The* katE* gene primers were designed using Primer3 software. All fold difference values were normalized with 23s rRNA expression and the values are the mean standard deviations; analysis is performed in triplicate.

Using bioinformatics prediction for chromosome 2, a potential RpoN box [4] was identified in front of the* katE* gene. An activity staining assay for catalase [14] was performed for the wild type* rpoN2* isogenic mutant and the* rpoN2* mutant carrying the* rpoN2-*complementing plasmid. The results suggest that catalase E or KatE activity is regulated by RpoN2 as shown in Figure 4(a). In particular, the* rpoN2* complemented strain fully restores the KatE activity as shown in Figure 4(a). To demonstrate that catalase E activity is regulated at the transcriptional level, qRT-PCR was performed as shown in Figure 4(b).* katE* expression in the wild type was detected from 48 hours to 72 hours of bacterial growth, whereas no* katE* expression was detected in the* rpoN2* mutant.

## 4. Discussion

In several bacterial pathogens, RpoN (*δ* ^54^) is known to be involved in pathogenesis and virulence such as nitrogen utilization and amino acid assimilation; capsular polysaccharide and lipopolysaccharide synthesis; flagella and pili biosynthesis; type III secretion; and biofilm formation [5–8, 22–24].* Rhizobium etli* has two copies of the* rpoN* gene and experiments with the mutant strains revealed that* rpoN1* and* rpoN2* genes are both active but under different physiological conditions [25]. The* rpoN1* gene is essential during the growth phase while* rpoN2* is required for metabolism [25]. In* B. pseudomallei*, each chromosome contains a member of the sigma 54 family (RpoN1 and RpoN2) which are known to be involved in amino acid utilization and some virulence factors but the function of each RpoN is still unclear. In this study, construction of both* rpoN1* and* rpoN2* knockout mutants was performed but, unfortunately, the* rpoN1* mutant could not be obtained. We therefore focused on the role of* B. pseudomallei* *δ* ^54^ (RpoN2) in comparison with the wild type strain PP844,* rpoN2* mutant, and complemented* rpoN2*. MM9 culture medium containing only inorganic salts, a carbon source, and water was used for testing whether RpoN2 was necessary for growth in the absence of amino acid supplementation. The wild type and the complemented* rpoN2* contain all the genes needed for survival and as predicted both of these strains can grow in the absence of the addition of exogenous amino acids (MM9). However, the* rpoN2* mutant is missing RpoN2 activity that has been proposed to regulate the transcription of some proteins involved in amino acid biosynthesis. If RpoN2 has a major role in amino acid utilization, the* rpoN2* negative mutant would be unable to grow in the absence of amino acid supplementation. Our results show that the* B. pseudomallei rpoN2* mutant strain was able to grow in MM9 medium indicating that RpoN2 is not essential in regulating amino acid utilization. However, the possibility that these phenotypes might be compensated by another* rpoN* gene product on chromosome 1 of* B. pseudomallei* could not be excluded.

In order to demonstrate the ability of* B. pseudomallei rpoN2* gene product to have a function in regulation of amino acid utilization, a complementation assay by restoration growth of* E. coli rpoN* mutant in minimal media supplemented with various amino acids (Table 2) was performed. Indeed, the ability to grow in the presence of glycine and methionine was the characteristic of* E. coli* wild type. Moreover, the ability to utilize glycine and methionine as sole nitrogen source in* E. coli* (Table 2) was previously shown to be regulated by RpoN [16, 23–27]. Due to the fact that all of the RpoN_Bp_ functional domains are almost identical to that of RpoN_Ec_, therefore, it is possible that RpoN_Bp_ is able to induce the transcription of the heterologous glycine and methionine utilization genes in* E. coli* JKD 814 in similar fashion to the* E. coli* RpoN.

In phenylalanine and glutamine utilization test, slower growth rate of JKD harboring TOPO::*rpoN2*_Bp_ compared to* B. pseudomallei* wild type was demonstrated (Table 2). It has been previously reported that the decrement in gene transcription by RpoN regulon is likely to be the consequence of the differences in the nucleotide sequence around the RpoN-conserved recognition sites 12 and 24 located upstream of the target genes [28]. Thus, it is possible that RpoN_Bp_ may prefer to recognize and induce the promoter sequence upstream of the genes required for phenylalanine and glutamine utilization in* B. pseudomallei* wild type more than that of* E. coli* JKD 814. However, it is also possible that phenylalanine and glutamine utilization system in* B. pseudomallei* wild type and* E. coli* may differ or operate differently. This hypothesis is also underscored by the data presented in Table 2 that* E. coli* wild type usually utilized phenylalanine and glutamine at least one day slower than* B. pseudomallei* wild type. Currently, the phenylalanine and glutamine utilization mechanisms in* B. pseudomallei* have not yet been elucidated. In addition, due to the difference in their native habitats, the discrepancy in the range of RpoN-controlled nitrogen utilization genes between* E. coli* and* B. pseudomallei* is also probably due to the specific control circuits to achieve the optimal adaptation to the different environments. The transport of nitrate across the membrane or the production of nitrate reductase and lysine decarboxylase in* B. pseudomallei* is not under the control of RpoN2_Bp_ as there was no different result observed among* E. coli* wild type,* E. coli* JKD 814, and JKD 814 derivatives. In* E. coli* and other Gram negative bacteria, lysine is metabolized by two major pathways, the lysine oxygenase and the pipecolate route [29, 30]. In the former route, L-lysine is oxidatively decarboxylated by lysine oxygenase to form 5-aminovaleramide, whereas in the latter, L-lysine is converted to D-lysine, which is then oxidatively deaminated, cyclized, and finally reduced to L-pipecolate [29, 30]. Moreover,* E. coli* can also utilize lysine as a sole carbon source and this ability is also under the control of *σ* ^54^ (RpoN) [29]. In contrast,* Pseudomonas putida*, the* B. pseudomallei* related species, was able to utilize lysine only as a nitrogen source and this ability was also under the control of *σ* ^54^ (RpoN) [15]. However, our results are not able to draw conclusion that the growth on minimal medium supplemented with lysine of* B. pseudomallei* and JKD 814 harboring TOPO::*rpoN2*_Bp_ is due to either the utilization of carbon or nitrogen source.

For better understanding the role of RpoN2 in amino acid utilization, we used 2-dimension polyacrylamide gels to identify proteins with altered levels in the* rpoN2* mutant since RpoN2 may regulate these proteins. Cells have many ways to obtain amino acids such as transport from environment,* de novo* synthesis, or synthesis from other amino acids and we speculated that RpoN2 might be involved in the regulation of some proteins involved in these processes. By analysis of soluble extracts using 2D gels and PDQuest software package to identify the proteins with altered abundance, we found that cysteine synthase (CysM) and phosphoribosyl formimino-5-aminoimidazole carboxamide ribotide isomerase (HisA) were both downregulated in the* rpoN2* mutant. While the levels of CysM and HisA were decreased in the* rpoN2* mutant, it was able to synthesize/take up sufficient cysteine and histidine to grow on MM9 medium. It is possible that both cysteine and histidine could be produced using intermediates from other pathways or other amino acids as precursors. Investigating the connections between cysteine synthesis and other biosynthetic pathways using http://www.genome.jp/kegg [13] revealed that in addition to the* de novo* pathway for cysteine synthesis it is possible to produce cysteine from glycine, serine, and threonine (Figure 2). The connection between cysteine synthesis and the production of other amino acids may explain why the* rpoN2* mutant, which should not be able to produce cysteine from the* de novo* pathway utilizing CysM, can grow on MM9 medium. As was seen with cysteine synthesis, histidine can also be synthesized from several precursors. HisA is a protein in amino acid metabolism specific to histidine biosynthesis and function in the synthesis of L-histidinol-P and purine metabolism from the pentose phosphate pathway as shown in Figure 3 (http://www.genome.jp/kegg) [13]. This protein was decreased in* rpoN2* mutant indicating that it is regulated by RpoN2; however alternative pathways are present that can compensate for loss of HisA dependent histidine production. Our study is the first to identify and demonstrate a role of RpoN2 in* B. pseudomallei*.

In contrast to the similarity of amino acid utilization between the* rpoN2* mutant and the wild type, the* rpoN2* mutant exhibited decreases in* katE* expression, both at the transcriptional and at the translational levels. Catalase is ubiquitous, well-studied enzyme that catalyzes the decomposition of hydrogen peroxide. It is an important enzyme for the survival of facultative aerobic organisms exposed to oxidative stress conditions. Using bioinformatics prediction for chromosome 2, a potential RpoN box [4] was identified in front of the* katE* gene. An activity staining assay for catalase [14] was performed. The results suggest that catalase E or KatE activity is regulated by RpoN2. In particular, the* rpoN2* complemented strain fully restores the KatE activity indicating that it is not due to polar effects on downstream genes. To demonstrate that the catalase E activity is regulated at the transcriptional level, qRT-PCR [11, 19, 20] was performed and the result is comparable to the KatE activity detecting from 48 hours to 72 hours of bacterial wild type growth. Interestingly, it has been reported that* B. pseudomallei* KatE activity [14] may be controlled by RpoS. In that study, the authors demonstrated the catalase activities at the translational level in which the catalase E activity was assessed only by activity staining assay. The contradictory results found in this study are more reliable because we determined that an RpoN box is in front of a* katE* gene and also because the* katE* gene expression was detected by qRT-PCR. We therefore hypothesized a possibility of cross communication between these two sigma families, RpoS and RpoN2, via KatE regulation, and that is currently under our investigation.

In conclusion, we report a novel finding that* B. pseudomallei* has 2 copies of RpoN and that RpoN2 is located on chromosome 2.* rpoN2* does not directly function in amino acid utilization in* B. pseudomallei* but it can restore this function in* E. coli*. The constructed* B. pseudomallei rpoN2* mutant lacking KatE activity is demonstrated by activity staining assay and qRT-PCR. In contrast to RpoN1, RpoN2 is therefore involved in a specific function for the regulation of catalase E expression both at the transcriptional and at the translational levels.

## Figures and Tables

**Table 1:** Bacterial strains and plasmids used in this study.

Strain or plasmid	Genotype or relevant characteristic	Source (reference)
Bacteria strains		
PP844	Wild type, clinical isolate from blood	This study
*E. coli* SM10 *λpir*	*λpir* (*thi thr leu tonA lacY supE recA::RP4-2-Tc::Mu Km*) used for transformation of recombinant plasmid pKNOCK*::rpoN2*_Bp_	[15]
*E. coli* JKD 814	*rpoN*::tet	[16]
Plasmids		
pKNOCK-Tc	Mobilizable suicide vector carrying Tet^R^ gene	[10]
pKRpoN2	pKNOCK-Tc containing 363 bp internal segment of *B. pseudomallei rpoN2* gene	This study
pBBR1MCS	Broad-host-range cloning vector, Cm^r^	[17]
pBSDRpoN2	pBR1MCS containing full-length *rpoN2* gene	This study
pCR 2.1-TOPO	Cloning vector for PCR product, Kanamycin and Ampicillin resistance	Invitrogen, California, USA
pTOPO::*rpoN2*_Bp799_	pCR 2.1-TOPO vector containing 799 bp fragment of truncated *rpoN2* gene from *B. pseudomallei* strain 844, Kanamycin and Ampicillin resistance	This study
pTOPO::*rpoN*_Ec_	pCR 2.1-TOPO vector containing full-length *rpoN* gene from *E. coli*, Kanamycin and Ampicillin resistance	This study

**Table 2:** Nitrogen and amino acid utilization of *B. pseudomallei* wild type 844 and *E. coli* derivatives.

Nitrogen source	Day of growth appearance (mean ± SD)^a^						
	*E. coli* JM 109	JKD 814 *rpoN* mutant	JKD 814 harboring pTOPO vector	JKD 814 harboring TOPO::*rpoN*_Ec_	JKD 814 harboring TOPO::*rpoN2*_Bp_	JKD 814 harboring TOPO::*rpoN2*_Bp799_	*B. pseudomallei* wild type 844
NH_4_Cl	3.7 ± 0.6^*∗*^	±	±	2.7 ± 0.6^*∗*^	2 ± 1	NG	4.7 ± 0.6^*∗*^
Arginine	1.3 ± 0.6^*∗*^	±	±	3.3 ± 0.6^*∗*^	3.3 ± 0.6^*∗*^	±	4.7 ± 0.6^*∗*^
Glutamine	2.7 ± 0.6^*∗*^	NG	NG	4.6 ± 0.6^*∗*^	3.7 ± 0.6^*∗*^	NG	1.3 ± 0.6^*∗*^
Glycine	2.7 ± 0.6^*∗*^	±	±	2.3 ± 0.6^*∗*^	2.7 ± 0.6^*∗*^	±	±
Histidine	1.3 ± 0.6^*∗*^	NG	NG	4 ± 1^*∗*^	2.3 ± 0.6^*∗*^	NG	1.3 ± 0.6^*∗*^
Lysine	3.3 ± 0.6^*∗*^	NG	NG	3.7 ± 0.6^*∗*^	4.3 ± 1.2^*∗*^	NG	4.7 ± 0.6^*∗*^
Methionine	4.7 ± 0.6^*∗*^	NG	NG	NG	4.7 ± 0.6^*∗*^	NG	±
Phenylalanine	4.7 ± 0.6^*∗*^	NG	±	4.7 ± 0.6^*∗*^	3.7 ± 0.6^*∗*^	±	1.3 ± 0.6^*∗*^
Tryptophan	1.7 ± 0.6^*∗*^	±	±	1.7 ± 0.6^*∗*^	1.7 ± 0.6^*∗*^	NG	2.7 ± 0.6^*∗*^
Valine	1.3 ± 0.6^*∗*^	NG	NG	NG	NG	NG	4.7 ± 0.6^*∗*^

^a^Data represent geometric mean (±standard error) from three independent experiments. NG indicates the absence of growth. ± indicates the very sparing growth inspected on day 6. ^*∗*^Significant differences in amino acid utilization compared to *E. coli rpoN* mutant JKD 814 (*P* ≤ 0.05, Student's paired *t*-test).

**Table 3:** Comparative partial proteomic analysis of *rpoN2* mutant compared to wild type of *B. pseudomallei.* The spot numbers assigned in this study are identical to the number of spots in *B. pseudomallei* 2DE reference map [18].

Protein orthologues	Expression alteration ratio in *B. pseudomallei *strains		pI	MW	Spot number
	Wild type	*rpoN2* mutant			
(1) Cell envelope biogenesis and outer membrane					
Acetyltransferase (GNAT) family protein	1	↑ 8.30	4.9	50.17	9
UDP-N-acetylmuramyl pentapeptide synthase	1	↑ 5.02	5.7	41.55	2
(2) Energy metabolism					
Polyphosphate kinase 2 family (Ppk2)	1	↑ 3.17	4.89	44.26	40
ATB synthase subunit B	1	↑ 3.40	4.97	53.93	15
NADH-dehydrogenase delta subunit	1	0.17 ↓	5.34	36.59	26
Aldehyde dehydrogenase (NAD) family protein	1	0.19 ↓	6.02	55.39	24
(3) Carbohydrate metabolism					
Glyceraldehyde 3-phosphate dehydrogenase (GapA)	1	↑ 3.38	4.82	20.88	48
Nucleoside-diphosphate-sugar epimerases	1	↑ 12.58	5.21	27.78	56
(4) Lipid metabolism					
4-Hydroxyl-3-methylbut-2-enyl diphosphate reductase (lspH)	1	↑ 5.07	5.94	40.81	71
Nonribosomally encoded peptide/polyketide synthase (CmaB)	1	0.21 ↓	6.19	37.19	73
(5) Amino acid metabolism					
Phosphoribosyl formimino-5-aminoimidazole carboxamide ribotide isomerase (HisA)	1	0.20 ↓	4.42	31	83
Cysteine synthase (CysM)	1	0.33 ↓	5.75	36.11	80
(6) Nucleotide metabolism					
Orotidine 5′-phosphate decarboxylase	1	↑ 6.42	4.99	32.79	88
(7) Translation					
50S ribosomal protein L3	1	↑ 3.49	4.14	27.8	150
(8) Transcription					
Transcriptional regulator, ArsR family 1	1	0.20 ↓	4.92	37.92	124
(9) Posttranslational modification					
Thioredoxin	1	↑ 3.37	5.97	16.15	179
Peptidyl-prolyl cis-trans isomerase B	1	↑ 22.15	6.05	19.99	174
Glutathione S-transferase	1	↑ 3.05	6.5	22	176
(10) Cell motility, intracellular trafficking, and secretion					
Type III effector protein BipD	1	↑ 5.04	4.38	34.37	194
Type III secretion chaperone LcrH/SycD	1	↑ 6.23	4.92	18.52	190
(11) Stress responses					
Carbohydrate porin, OprB family	1	↑ 3.16	5.01	38.71	233
(12) Hypothetical protein					
Hypothetical protein BPSL0349	1	↑ 3.15	5.28	16.53	253
Hypothetical protein BPSS0931	1	0.03 ↓	4.64	23.63	274

## Abbreviations

*B. pseudomallei*: * Burkholderia pseudomallei*
2DE: Two-dimensional gel electrophoresis
KatE: Catalase E
qRT-PCR: Quantitative Reverse Transcriptase-Polymerase Chain Reaction.
